# Three-dimensional (3D) ultrasound imaging for quantitative assessment of frontal cobb angles in patients with idiopathic scoliosis – a systematic review and meta-analysis

**DOI:** 10.1186/s12891-025-08467-5

**Published:** 2025-03-05

**Authors:** Cheuk-Kin Kwan, James Haley Young, Jeff Ching-Hei Lai, Kelly Ka-Lee Lai, Kenneth Guang-Pu Yang, Alec Lik-Hang Hung, Winnie Chiu-Wing Chu, Adam Yiu-Chung Lau, Tin-Yan Lee, Jack Chun-Yiu Cheng, Yong-Ping Zheng, Tsz-Ping Lam

**Affiliations:** 1 SH Ho Scoliosis Research Lab, Joint Scoliosis Research Center of the Chinese University of Hong Kong and Nanjing University, Hong Kong, Hong Kong; 2 Department of Biomedical Engineering, The Hong Kong Polytechnic University, Hong Kong, Hong Kong; 3 Department of Imaging and Interventional Radiology, The Chinese University of Hong Kong, Hong Kong, Hong Kong

**Keywords:** Scoliosis, Ultrasound, Adolescent Idiopathic Scoliosis

## Abstract

**Background:**

Measurement of Cobb angle in the frontal plane from radiographs is the gold standard of quantifying spinal deformity in adolescent idiopathic scoliosis (AIS). As a radiation free alternative, ultrasonography (USG) for quantitative measurement of frontal cobb angles has been reported. However, a systematic review and meta-analysis on the reliability of ultrasound comparing with the gold standard have not yet been reported.

**Objectives:**

This systematic review and meta-analysis aimed to evaluate (1) the reliability of ultrasound imaging compared with radiographs in measuring frontal cobb angle for screening or monitoring in AIS patients; (2) whether the performance of USG differ when using different anatomical landmarks for measurement of frontal cobb angles.

**Methods:**

Systematic search was performed on MEDLINE, EMBASE, CINAHL, and CENTRAL databases for relevant studies. QUADAS-2 was adopted for quality assessment. The intra- and inter-rater reliability of ultrasound measurement in terms of intra-class correlation coefficient (ICC) was recorded. Mean Absolute Difference (MAD) and Pearson correlation coefficients between frontal cobb angle measured from USG and radiographic measurements, were extracted with meta-analysis performed.

**Results and discussion:**

Nineteen studies were included with a total of 2318 patients. The risk of bias of included studies were unclear or high. Pooled MAD of frontal cobb angle measured between USG and radiography was 4.02 degrees (95% CI: 3.28–4.76) with a pooled correlation coefficient of 0.91 (95% CI: 0.87–0.93). Subgroup analyses show that pooled correlation was > 0.87 across using various USG landmarks for measurement of frontal cobb angles. There was a high level of heterogeneity between results of the included studies with I^2^ > 90%. Potential sources of heterogeneity include curve severity, curve types, location of apex, scanning postures, patient demographics, equipment, and operator experience. Despite being the “gold standard”, intrinsic errors in quantifying spinal deformities with radiographs may also be a source of inconsistency.

**Conclusion:**

The current systematic review indicated that there is evidence in favor of using USG for quantitative evaluation of frontal cobb angle in AIS. However, the quality of evidence is low due to high risk of bias and heterogeneity between existing studies. Current literature is insufficient to support the use of USG as a screening and/or follow-up method for AIS. Further investigation addressing the limitations identified in this review is required before USG could be adapted for further clinical use.

**Supplementary Information:**

The online version contains supplementary material available at 10.1186/s12891-025-08467-5.

## Introduction

Measurement of Cobb angle in the frontal plane from radiographs is the gold standard of quantifying spinal deformity in adolescent idiopathic scoliosis (AIS). However, radiation from repeated radiography poses health concerns for patients [1, 2]. To reduce radiation hazards, alternative imaging methods have been investigated for quantitative spinal assessment [3]. Among these imaging modalities, ultrasound imaging carries significant advantages of being a well-established, radiation-free, cost-effective, and portable method capable of dynamic scanning. As such, the application of ultrasound imaging in musculoskeletal diagnostics has gained considerable attention over the past decade [4–7].

**Fig. 1:**
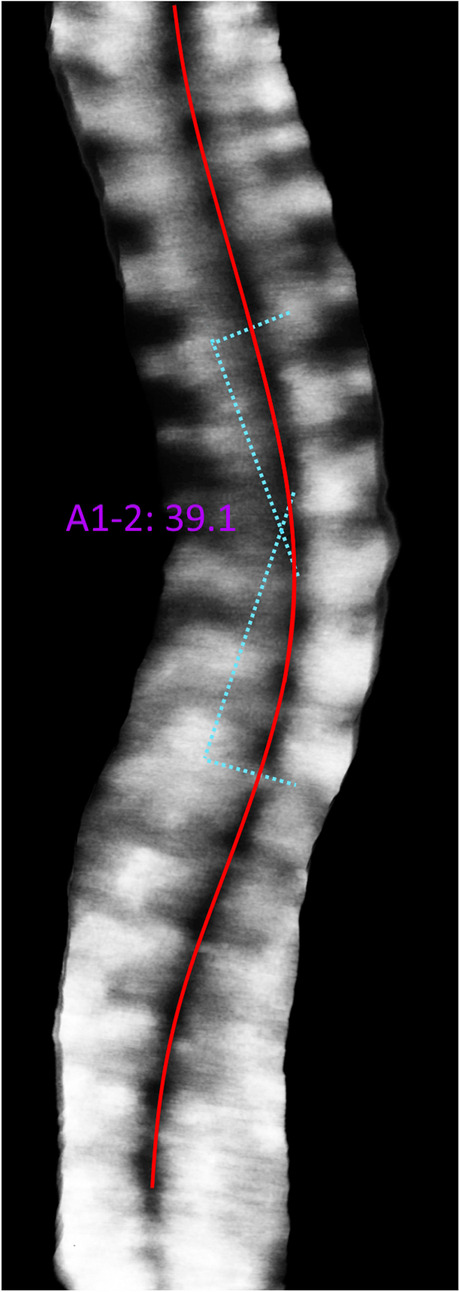
Spinous process angles (SPA) measurement on an ultrasound image

**Fig. 2:**
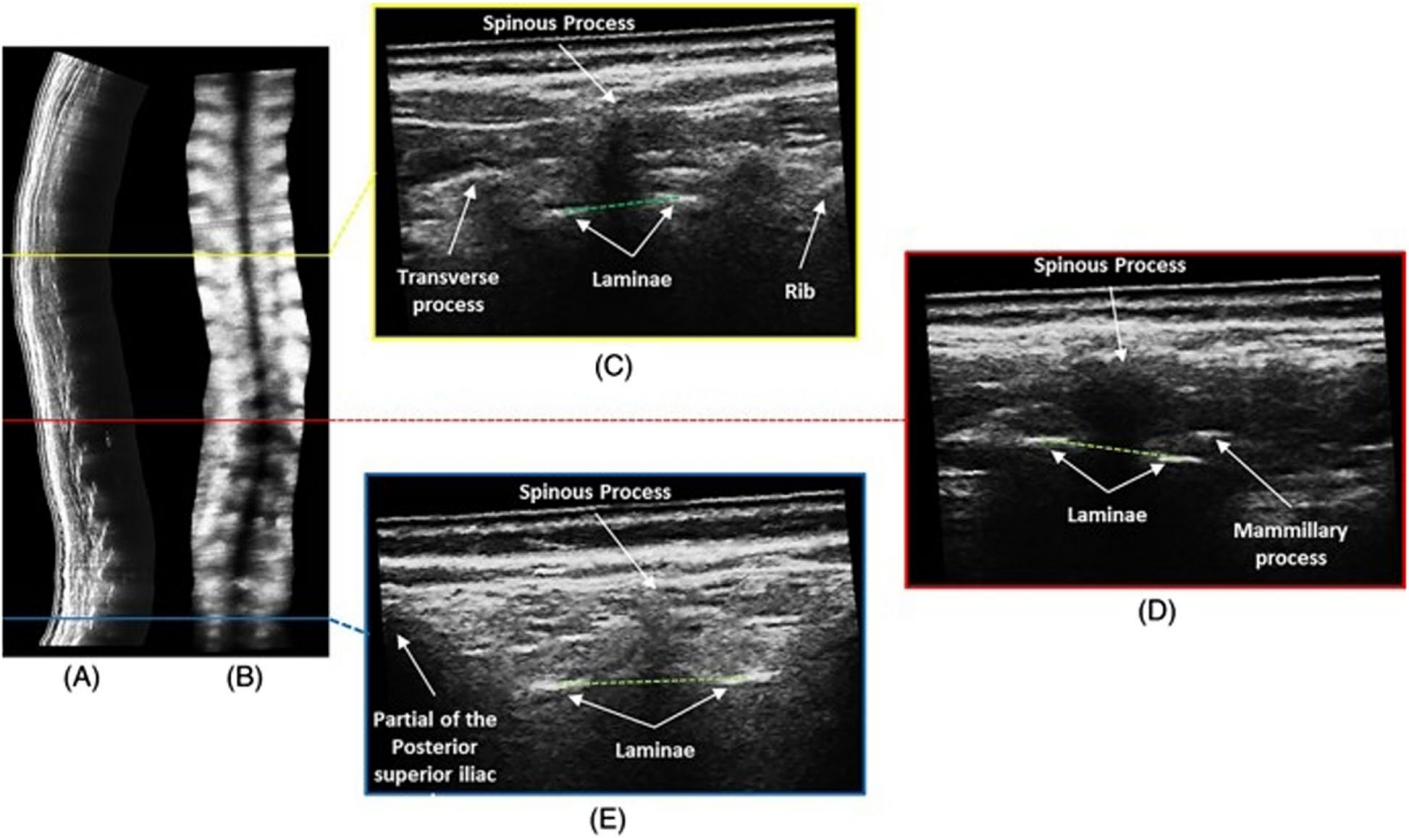
Center of lamina (COL) method for measurement of coronal curvatures and axial vertebral rotation on reconstructed 3D ultrasound images. Adopted with permission under Creative Commons Attribution License “Three-dimensional Ultrasonography Could be a Potential Non-ionizing Tool to Evaluate Vertebral Rotation of Subjects with Adolescent Idiopathic Scoliosis” by Lee et al. [23]. a Sagittal plane. bCoronal plane. Corresponding transverse plane at: c T8 level. d L1 level. e S1 level. The green dotted lines join the laminae at the 3 above mentioned levels

**Fig. 3:**
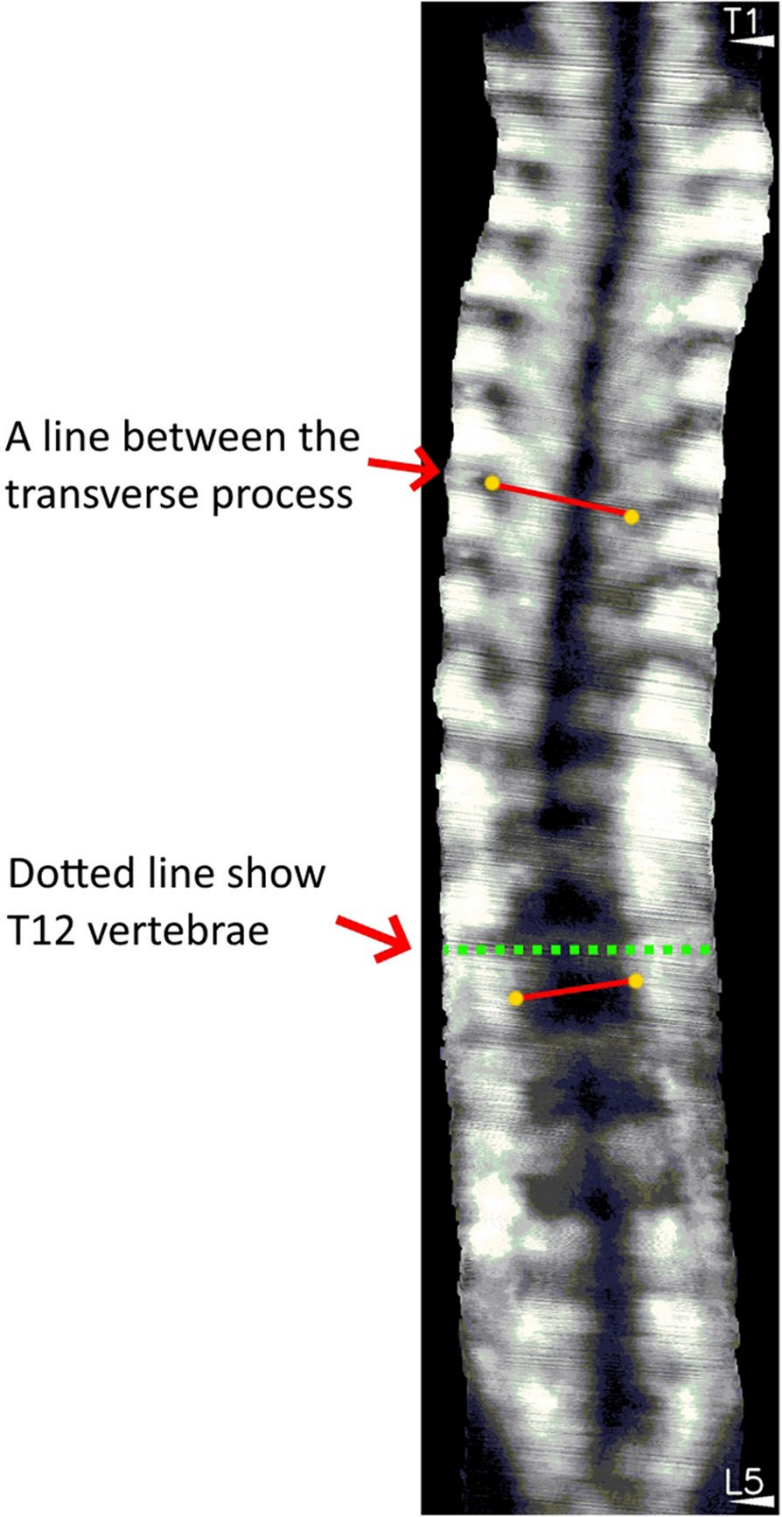
Transverse processes angle (TPA) measurement on coronal ultrasound image

As the cortical surface of bone strongly reflects ultrasound waves to generate a bony shadow in B-mode images, ultrasound imaging can be used to detect the posterior arch of the spine, and display the rotatory position of laminae and transverse processes for the measurement of vertebral rotation [8–11]. With the development of freehand three-dimensional ultrasound imaging systems that combine conventional B-mode images with position sensors for a three-dimensional reconstruction of full spine images, the limitation of a previously two-dimensional image can be overcome, which is crucial to analyzing the three-dimensional anatomy of each spine [12, 13]. The validity and reliability of ultrasound evaluation based on the anatomical landmarks of spinous processes (SP) [5, 6], transverse processes (TP) [5, 12], and laminae [10, 14] for ultrasound measurement of spinal curvatures in the coronal, sagittal, and transverse planes have been reported in both in vitro [15–20] and in vivo studies [5, 6, 14, 21, 22] (Figs. 1, 2 and 3) [23]. Despite promising results, many of the three-dimensional ultrasound imaging systems were experimental prototypes that were not optimized for large-scale clinical application [15–18, 24, 25]. In vitro results are deemed to be more accurate than in vivo measurements, as patients’ posture was not taken into account in in vitro studies [12, 18]. Cadaveric bony landmarks, particularly the laminae, are also much more easily identified on ultrasound imaging when compared with that for living subjects, especially for those with a high body mass index (BMI) [11, 26]. To evaluate the use of ultrasound imaging for living subjects, a number of in vivo studies have been conducted [5, 6, 14, 21, 22]. Nevertheless, ultrasound imaging for scoliosis assessment is still in a developmental stage [6]. No conclusive statement has yet been drawn regarding how ultrasound imaging can serve as an alternative imaging modality for spinal evaluation in an accurate and reliable manner to minimize radiation exposure in adolescents. In addition, it remained controversial regarding which anatomical landmarks could provide the best estimation of frontal cobb angles [5, 27].

We therefore carried out a systematic review with meta-analysis to evaluate (1) the intra- and inter-rater reliability of ultrasound measurement and its validity in terms of correlation and mean absolute difference (MAD) with the gold standard of radiological frontal plane Cobb angle in measuring spinal deformity in AIS patients; (2) whether the correlation of ultrasonography (USG) with radiological frontal Cobb angle differed when using different anatomical landmarks for measurement of spinal curvatures.

## Materials and methods

### Information sources and searching strategy

Relevant studies that involved ultrasound imaging for quantitative assessment of the spine were searched from four databases, namely MEDLINE, EMBASE, CINAHL, and Cochrane Library (CENTRAL) databases. The Preferred Reporting Items for Systematic Reviews and Meta-Analyses for Diagnostic Test Accuracy studies (PRISMA-DTA) statement was used as guidelines in the performance of the systematic review. The search was limited to English publications up to 31 December 2023. Specific searching strategies adapted for each database were listed in Appendix I. The reference lists of all included studies were also examined for additional relevant studies.

### Selection of studies

Articles were included if they met the following criteria:

1. Clinical trials, observational studies, or diagnostic accuracy studies, which reported on the error AND/OR correlation between ultrasound imaging and radiographic measurement of frontal cobb angles in patients with adolescent idiopathic scoliosis (AIS)
2. Full publication in a peer-reviewed scientific journal

The exclusion criteria were:

1. *In* *vitro* experiments, phantom studies, or pilot studies involving < 10 patients
2. Non-adolescent idiopathic scoliosis subjects
3. Application of ultrasound imaging other than quantification of frontal cobb angles, for example, muscle quantification, skeletal maturity assessment, bone quality measurement, spinal flexibility measurement, brace casting, orthotic design, surgical related procedures like anesthesia, operative guidance, or imaging for magnetically controlled growing rods
4. Review articles, editorials, letters, comments, case reports, or conference abstracts
5. Non-English studies

Studies from the systematic search were merged in EndNote X9 (Thomson, New York), with duplicates removed. Application of exclusion and inclusion criteria was performed by screening the titles and abstracts, followed by retrieval of full texts of included studies. Two reviewers (JHY, JCL) independently screened all the titles and abstracts, and reviewed the identified studies for inclusion. Disagreements were resolved by consensus between the 2 reviewers. A third reviewer (KGY) was available to resolve further disagreements.

### Quality assessment

Risk of bias and concerns regarding applicability of each included study were evaluated using the Quality Assessment of Diagnostic Studies-2 (QUADAS-2) instrument (Appendix II) [28]. Radiographic measurement was designated as the “reference standard” and ultrasound measurement was defined as the “index test”. The assessment of study quality was performed in a standardized manner independently by two reviewers (JHY, JCL). Disagreements were resolved by consensus between the 2 reviewers. A third reviewer (KGY) was available to resolve further disagreements.

### Data extraction and meta-analysis

The intra- and inter-rater reliability of ultrasound measurement in terms of intra-class correlation coefficient (ICC) was recorded. If more than one ICC value was reported for different raters or per different scans in the articles, the lowest value was recorded. MAD and Pearson correlation coefficients (r) between ultrasound imaging and radiographic measurement were extracted and reanalyzed to obtain the pooled correlation and 95% confidence intervals (CIs) using the random effects model of meta-analysis and presented as a forest plot. If more than one correlation coefficient value was reported for various curve location, they were considered independently. Heterogeneity across studies was tested by the inconsistency index (I^2^) [29]. Two subgroup analyses were performed according to the ultrasound measurement protocols adopted, namely the spinous process method, transverse processes (TP) method, and center of lamina (COL) method.

MedCalc® Statistical Software version 20.305 (MedCalc Software Ltd, Ostend, Belgium) was used. *p* < 0.05 was considered statistically significant.

## Results

### Literature search and selection of studies

**Fig. 4:**
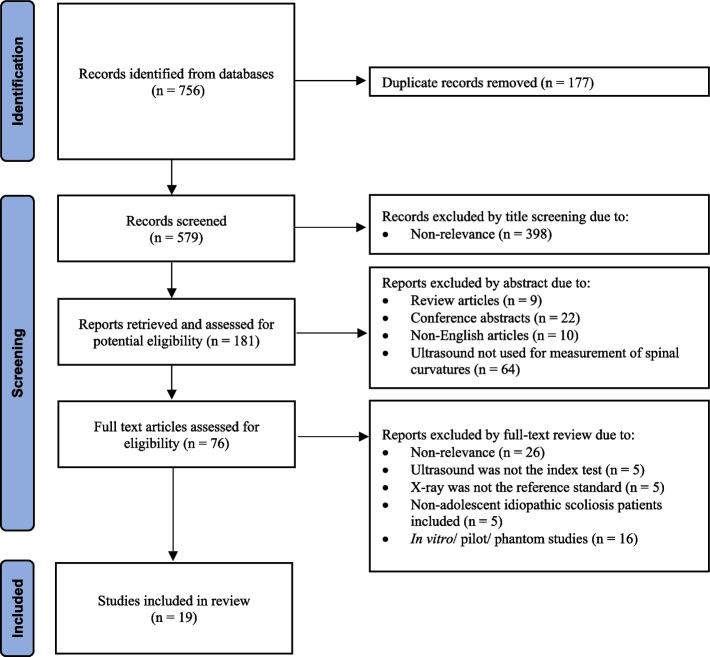
The PRISMA selection flow diagram

Five hundred seventy-nine studies were identified after removal of duplicates, of which 398 studies were initially excluded because of non-relevance (Fig. 4). 181 potentially eligible studies were examined in full text. Eventually, 19 articles met the selection criteria and were included for meta-analysis [5, 6, 8, 11–14, 30–41].

The 19 included articles were published between 2015 and 2022 with a total of 2318 participants. Among included studies, 6 explored the COL method, 5 explored the TP method, and 12 explored the spinous process method. Out of 19 included studies, intra-rater reliability was reported in 15, while inter-rater reliability was reported in 11 studies for USG measurement of frontal cobb angle. MAD between USG and radiography measured frontal cobb angle was reported in 13, while Pearson correlation coefficient was reported in 16 studies. The data extraction table is presented as Table 1.

**Table 1:** Data extraction table. NA = Not applicable

**First author**	**Journal**	**Coronal radiological cobb angles (Mean ± SD, range)**	**Sub-group**	**Mean absolute difference/root mean square (degrees)**	**SD (degrees)**	**Ultrasound measurement protocol(s)**	**Intra-rater reliability of ultrasound measurement (ICC)**	**Inter-rater reliability of ultrasound measurement (ICC)**	**Pearson correlation coefficient between coronal ultrasound and radiographic measurements**	**Origin**
Zheng (2018) [14]	PLoS One	23.7 ± 9.5, 10.0–53.0		2.1	1.7	COL	0.96	Not Given	0.959	Canada
Brink (2018) [5]	The Spine Journal	Thoracic 38.5 ± 20.4, 6.0–90.0; Lumbar 28.9 ± 11.3, 3.0–52.0	automatic SP angle	4.9	3.2	SP; TP	Automatic SP 0.97	Automatic SP 0.94	Automatic SP 0.991;	The Netherlands
			manual SP angle	4.5	3.1		Manual SP 0.96	Manual SP 0.86	Manual SP 0.985	
			manual TP angle	4.7	3.6		Manual TP 0.94	Manual TP 0.84	Manual TP 0.992	
Zheng (2016) [6]	Spine (Phila Pa 1976)	24.8 ± 9.0, 10–46		4.7	n/a	COL	AOR method 0.94; Blinded measurement 0.86	AOR method 0.91; Blinded measurement 0.83	AOR method 0.92; Blinded measurement 0.76	Canada
Zheng (2015) [31]	Spine Deformity	23.6 ± 7.0, 12.0–45.0		3.5	2.4	COL	0.84	0.8	0.78	Canada
Cheung (2015) [12]	Journal of Orthopaedic Translation	1.9–29.9		n/a	n/a	TP; TP-SAP (Transverse process-superior articular process)	TP 0.57; TP-SAP 0.93	TP 0.75; TP-SAP 0.89	TP (*n* = 27) 0.825; TP-SAP (*n* = 28) 0.927	Hong Kong
Zheng (2016) [30]	Scoliosis and Spinal Disorders	24.8 ± 9.7, 3.0–48.0		2.8	n/a	SP	0.88	0.87	0.873	Hong Kong
Zhou (2017) [11]	IEEE Transactions on Medical Imaging	1.9–29.9		n/a	n/a	SP	Not Given	Not Given	0.83	Hong Kong
Cheung (2015) [13]	IEEE Transactions on Medical Imaging	1.9–29.9		1.9	7.3	SP; TP	SP 0.99; TP 0.98	SP 0.92; TP 0.96	SP 0.889; TP 0.883	Hong Kong
Li (2015) [8]	Spine Deformity	20.0–40.0		n/a	n/a	SP	0.91	Not Given	0.792	Hong Kong
Lee (2019) [33]	Ultrasound in Medicine and Biology	24.5 ± 9.0	USSPA	6.1	4.4	SP; COL	0.92	0.94	NA	Hong Kong
			USLA	5.3	4.2					
Yang (2022) [34]	Spine Deformity	25.5 ± 9.6	Thoracic	3.6	2.5	TP	Not Given	Not Given	Thoracic 0.887; Lumbar 0.926	Hong Kong
			Lumbar	3.4	2.9					
Pang (2021) [35]	Ultrasound in Medicine and Biology	Not Given		n/a	n/a	SP	0.988	0.949	0.819	Hong Kong
Wong (2019) [36]	Ultrasound in Medicine and Biology	29.3 ± 11.8		n/a	n/a	SP	0.988	0.949	0.816	Hong Kong
Lee (2021) [37]	Journal of Orthopaedic Translation	Thoracic 25.1 ± 9.6; Lumbar 24.0 ± 9.1	Thoracic	3.0	1.5	TP + SP	0.973	0.925	Thoracic 0.94; Lumbar 0.945	Hong Kong
			Thoracolumbar	2.8	1.4					
Young (2015)[38]	European Spine Journal	22.4 ± 7		2.6	2.0	COL	0.89	n/a	n/a	Canada
Trac (2018) [32]	Spine Deformity	26 ± 9		3.2	2.2	COL	0.9	n/a	n/a	Canada
de Reuver (2021) [39]	European Spine Journal	38.4 ± 20.5	Thoracic	6.5	3.9	SP	n/a	n/a	0.959	Netherlands
		26.3 ± 13	Lumbar	7.3	4.7		n/a	n/a	0.936	
Trzcinska (2022) [40]	International journal of environmental research and public health	32.45 ± 6.53		n/a	n/a	SP	n/a	n/a	0.7	Poland
Zeng (2021) [41]	IEEE transactions on ultrasonics, ferroelectrics, and frequency contro	n/a		5.8	4.9	SP	0.95	0.93	0.90	Shanghai

**First author**	**Sample size (Males/Females)**	**Age (Mean ± SD, range)**	**Participants**	**Ultrasound system**	**Ultrasound Probe frequency and shape**	**Delineation**	**Scanning posture**	**Investigated levels**	**Time interval between ultrasound and radiographic examination**
Zheng (2018) [14]	200 (30/170)	14.6 ± 1.9, 10.2–18.3	JIS or AIS patients	SonixTABLET ultrasound system	3.5 MHz curved	Semi-automatic + aid of previous radiographs (AOR)	Standing	C7 to L5	Same day
Brink (2018) [5]	33 (3/30)	13.8 ± 2.3, 10.0–17.0	AIS patients	Scolioscan	7.5 MHz linear	Automatic and manual SP; Manual TP	Standing	T1 to S1	Same day
Zheng (2016) [6]	65 (11/54)	14.7 ± 1.9	AIS patients	SonixTABLET ultrasound system	3.5 MHz curved	Manual ± aid of previous radiographs (AOR)	Standing	C7 to L5	Same day
Zheng (2015) [31]	26 (4/22)	13.9 ± 2.1	AIS patients	SonixTABLET ultrasound system	3.5 MHz curved	Manual	Standing	C7 to L5	Not same day
Cheung (2015) [12]	28 (9/19)	28.0 ± 13.0	Volunteers	Ultrasound scanner EUB-8500	7.5 MHz linear	Manual	Standing	T1 to L5	< 3 months
Zheng (2016) [30]	49 (15/34)	15.8 ± 2.7, 11.0–23.0	AIS patients	Scolioscan	7.5 MHz linear	Semi-automatic	Standing	T1 to L5	Not same day
Zhou (2017) [11]	29 (9/20)	30.6 ± 14.7, 10.0–52.0	Volunteers	Scolioscan	7.5 MHz linear	Automatic	Standing	Not Given	Not same day
Cheung (2015) [13]	29 (9/20)	30.6 ± 14.7, 10.0–52.0	Volunteers	Ultrasound scanner EUB-8500	7.5 MHz linear	Manual	Standing	T1 to L5	Not Given
Li (2015) [8]	33 (0/33)	9.0–14.0	AIS patients	Esaote Technos MPX clinical ultrasound unit	7.5 MHz linear	Manual	Standing	C7 to L5	Same day
Lee (2019) [33]	21 (7/14)	15.7 ± 1.3	AIS patients	Scolioscan	7.5 MHz linear	Manual	Standing	C7 to L5	Same day
Yang (2022) [34]	100 (19/81)	15.0 ± 1.9	AIS patients	Scolioscan	7.5 MHz linear	Semi-automatic	Standing	Not Given	Same day
Pang (2021) [35]	442 (199/243)	13.2 ± 1.8	AIS patients	Scolioscan	7.5 MHz linear	Automatic	Standing	T1 to L5	Same day
Wong (2019) [36]	952 (231/721)	16.7 ± 3.0	AIS patients	Scolioscan	7.5 MHz linear	Automatic	Standing	T1 to L5	Same day
Lee (2021) [37]	50 (11/39)	15.2 ± 1.9	AIS patients	Scolioscan	7.5 MHz linear	Manual	Standing	T1 to L5	Same day
Young (2015)[38]	20 (4/16)	14.5 ± 1.7	AIS patients	SonixTABLET US	3.5 MHz curved	Manual	Standing	C7 to L5	Same day
Trac (2018) [32]	101 (14/87)	13.7 ± 1.7	AIS patients	SonixTABLET	3.5 MHz curved	Manual	Standing	C7 to L5	Same day
de Reuver (2021) [39]	70 (14/56)	14.5 ± 2	AIS patients	Scolioscan	7.5 MHz linear	Manual	Standing	T1 to S1	Same day
Trzcinska (2022) [40]	20 (0/20)	14.05 ± 1.64	AIS patients	Scolioscan	7.5 MHz linear	Manual	Standing	T1 to L5	n/a
Zeng (2021) [41]	50 (n/a)	n/a	AIS patients	n/a	/	Automatic	Standing	n/a	n/a

### Quality assessment

Twelve out of 19 studies showed low levels of concern across all domains regarding applicability, indicating that the study designs match with the review question. However, only 1 out of 19 studies demonstrated a low risk of bias, with the remaining 18 studies showing an unknown or high risk in at least one domain in the QUADAS-2 assessment tool. Details of quality assessment are displayed in Tables 2 and 3.

**Table 2:**
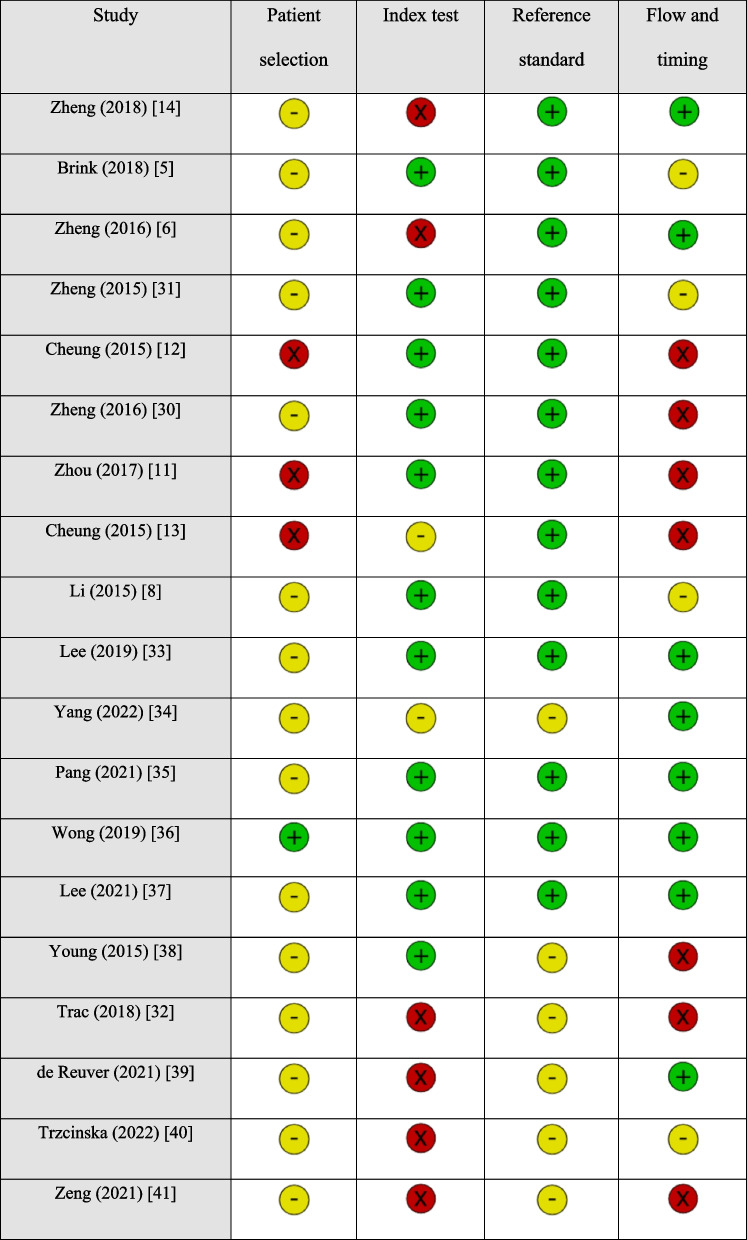
Risk of bias (RoB) of each included study [5, 6, 8, 11–14, 30–41]

**Table 3:**
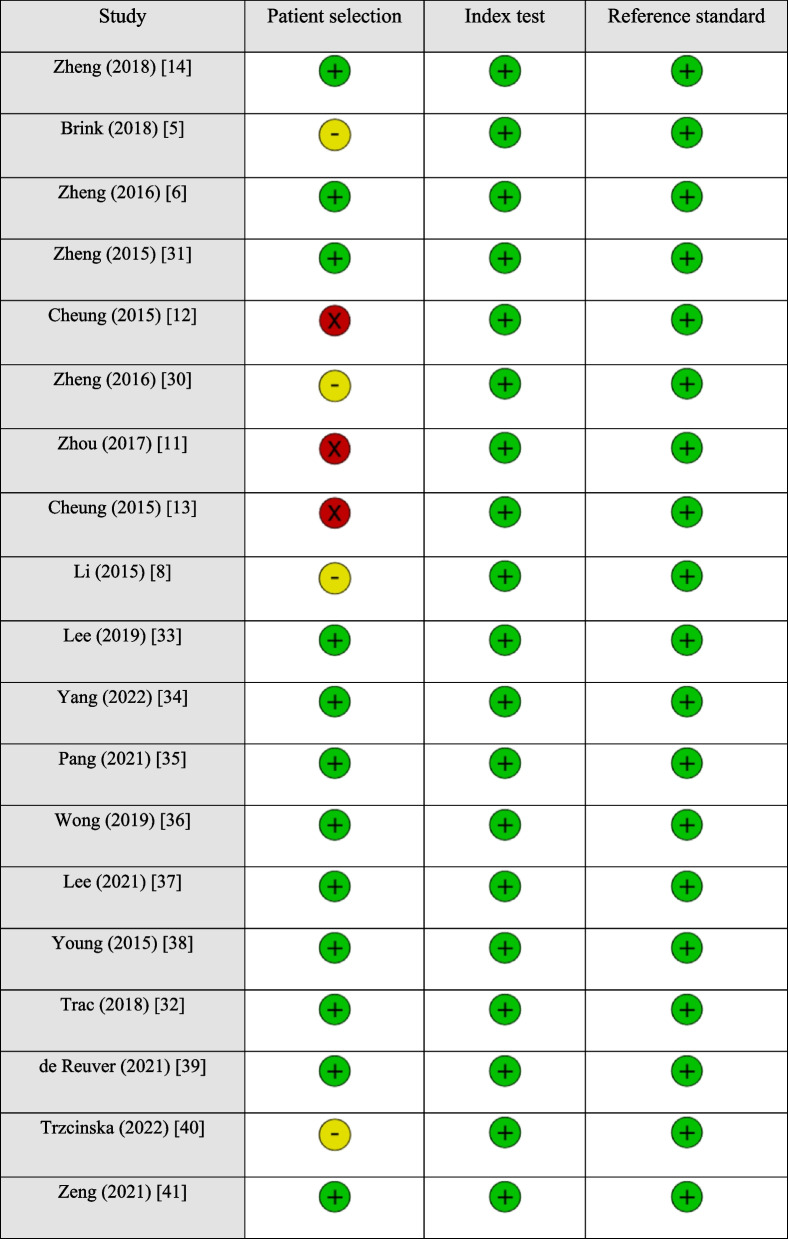
Applicability concerns of each included study [5, 6, 8, 11–14, 30–41]

### Data extraction and meta-analysis

Intra-rater reliability of ultrasound measurement ranged from 0.57 to 0.99 (mean 0.96 ± 0.06); whereas the inter-rater reliability ranged from 0.75 to 0.96 (mean 0.93 ± 0.04).

**Fig. 5:**
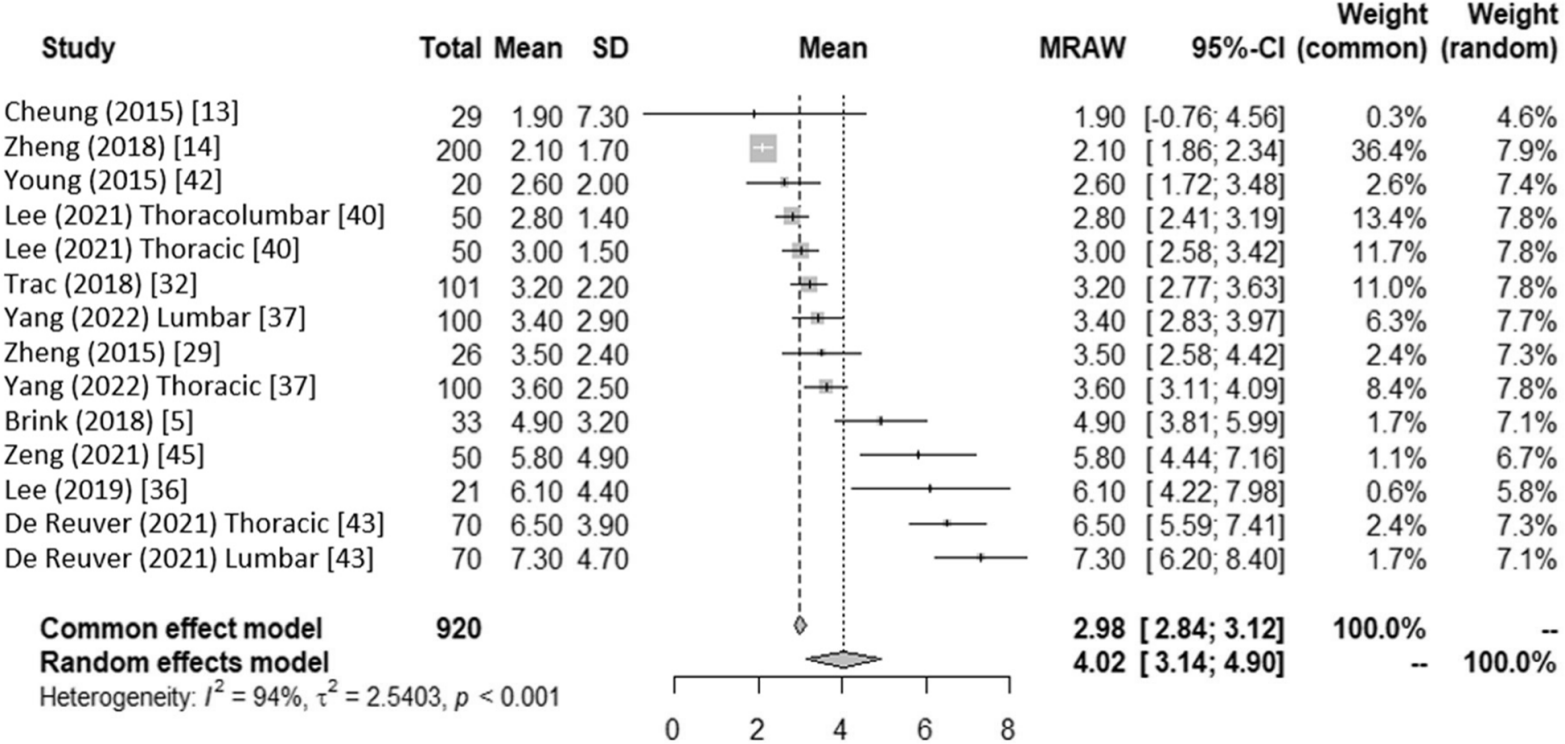
Forest plot of the Mean Absolute Difference between ultrasound and radiographic measurement with corresponding 95% CIs for studies on the coronal plane. If two correlation analyses were drawn from the same method *and* plane in the same study, the difference is denoted within the bracket. E.g., Lee (2021) [40] has separate correlation analyses for thoracic and lumbar curves, although both evaluated the coronal plane with the spinous process method

Meta-analysis showed the pooled MAD in frontal Cobb angle measurements when comparing radiographs versus ultrasound was 4.02 degrees (95% CI:3.28–4.76, I^2^ = 94%) (Fig. 5). Pooled Pearson correlation coefficient for frontal Cobb angle measurements when comparing radiographs versus ultrasound was 0.91 (95% CI: 0.87–0.93, I^2^ = 90%).

Subgroup analyses on the various ultrasound measurement protocols showed that the pooled correlations were 0.87 (95% CI: 0.72–0.93, I^2^ = 92%) for the center of lamina (COL); 0.90 (95% CI: 0.85–0.93, I^2^ = 90%) for the spinous process (SP) method; and 0.94 (95% CI: 0.88–0.97, I^2^ = 90%) for the transverse process (TP) method.

## Discussion

Results from this study showed good intra- and inter-rater reliability of ultrasound measurement of frontal cobb angle in AIS patients. It also showed good validity in terms of correlation and MAD when compared with the gold standard of radiological frontal cobb angle in AIS patients. However, the included studies demonstrated unclear to high risk of bias. The strengths and limitations of the included studies and the current methods of ultrasound measurement of frontal cobb angles in AIS patients will be discussed in the following section.

### Strengths and limitations of different methods of ultrasound

The spinous process (SP) method was the most frequently adopted protocol overall (12 out of 19 studies). However, previous studies have reported limitations of the spinous process method. Significant deviation of spinous processes due to significant axial vertebral rotation would also cause inaccurate interpretation of the vertebral body alignment and hence influence the angle measurement on ultrasound imaging [6, 11, 12, 22, 42]. Various conversion formulae have been proposed for systematic correction of the spinous process method values for prediction of Cobb angles based on relatively small cohorts of subjects [5, 6, 43]. In future studies, establishment of conversion formulae from large-scale studies with adjustment according to various curve levels and curve severity would be warranted.

The center of lamina (COL) method was another commonly used approach (6 out of 19 studies). However, the pooled correlation with this approach against radiographic measurements was the weakest among the three methods compared (*r* = 0.86). Theoretically, the close relationship between the laminae assessed in the COL method and the vertebral bodies used in calculating the Cobb angle on radiography would allow more accurate estimation [26]. However, in practice, the laminae are also located deeper than the spinous processes along the posterior-anterior direction and are therefore more difficult to detect, even more so in the lumbar region and in obese subjects [26, 31].

The strongest level of correlation (*r* = 0.94) was demonstrated by the transverse processes (TP) method, possibly because the vertebral bodies used in radiographic Cobb measurement were along the same direction of the lines connecting the pairs of transverse processes [13]. However, widespread application of this method is limited by the difficulty in visualizing the transverse processes on ultrasound images [13]. As transverse processes are located beneath the thick and unevenly distributed paraspinal muscles at various depths, it is technically difficult to capture high quality ultrasound images even with the currently optimized default ultrasound setting of depth, focus, and frequency [13]. To add to the challenges of the TP method, an ultrasound probe with adequate width is required to cover all transverse processes from the spine in a single motion, and the view could also be obstructed by the winged scapula of scoliosis patients [6, 12, 13].

Given that various vertebral landmarks can be identified on ultrasound images, the use of combined landmarks to provide more anatomical information for ultrasound measurement has been explored [12, 37]. In particular, superior articular processes (SAP) have been used along with transverse processes to achieve better validity and reliability of coronal ultrasound measurement [12], but further clinical studies are warranted to explore the combination of various bony landmarks for better visualization of the spine to improve the accuracy of ultrasound angle measurement [6, 44].

### Sources of bias of included studies

Only 1 out of 19 studies demonstrated a low risk of bias, with the remaining 18 studies showing an unknown or high risk in at least one domain in the QUADAS-2 assessment tool.

Potential sources of bias include: (1) Unclear description of the recruitment process, whether a consecutive or random sampling was performed for patient recruitment. (2) the inclusion of subjects without scoliosis (Cobb angles < 10°) [5, 6, 11–13]; (3) exclusion of patients owing to missing data of radiographic assessment [5, 11, 13]; and (4) exclusion of patients with poor ultrasound image quality or unclear anatomical landmarks [5, 6, 12, 31], high BMI [6, 11], winged scapula [5, 6, 11], severe spinal curvatures [6, 14, 30, 31], or pre-selected patients with specific curve types [22, 27]. Exclusion of these “difficult to diagnose” subjects may lead to overestimation of the diagnostic accuracy of ultrasound imaging.

In addition, studies utilizing the aid of previous radiographs by overlaying them onto new ultrasound images significantly improved the accuracy and reliability of ultrasound measurement, owing to better guidance on identification of anatomical landmarks and reduction of variation in end-vertebra selection [31]. For valid comparison across various included articles, ultrasound imaging results should have been interpreted without being guided by past radiographic measurements. In addition, a delay of a week to even 3 months between ultrasound imaging and radiographic examinations were present in some of the included studies, which may have contributed to bias due to progression of scoliosis, postural change of the patients, or the corrective effect of brace treatment.

### Inconsistencies in radiographic Cobb angle measurements

Despite being the current “gold standard” of quantifying the frontal cobb angle in AIS patients, previous studies have made an argument that intrinsic errors exist in radiographic measurement.

Various factors have been suggested to contribute to the variability of such measurements, including but not limited to radiographic markers of wide diameter, selection of end vertebrate, observer bias, protractor accuracy, image acquisition techniques and time, image size, and positioning [45]. It is generally agreed that 5° is accepted as measurement variation between assessments [46]. Intra-observer variation 3–5° and inter-observer variation 6–9° have also been reported in the measurement of the Cobb angle [47–50]. Therefore, it may be important to acknowledge that inconsistencies observed between the use of ultrasonography versus traditional radiography for the quantification spinal deformities could be arising from an inconsistent “gold standard”.

### Limitations and future studies

Nineteen included studies were mostly preliminary studies confined to relatively small sample sizes, from only a few research groups. Included studies are also often from a small number of research groups, and there is also high heterogeneity among included studies.

Most included studies were published in Hong Kong and Canada, by the same groups of researchers. There is a possibility that included studies shared parts of the same cohorts as subjects.

In terms of heterogeneity, the I^2^ value was greater than 90% in the meta-analyses performed in this study. Keeping in mind the high heterogeneity in the results with unknown bias for the majority of the studies, the results from meta-analysis should be interpreted with caution. Potential sources of heterogeneity may be attributed to: (1) different curve severity; (2) different curve types and locations; (3) different scanning postures; (4) patient demographics; (5) different quality of equipment; and (6) experience of the operators. Given that further subgroup analyses on these parameters were limited owing to insufficient sample sizes available in the current literature, future studies that investigate the accuracy of ultrasound measurement in relation to these different parameters are warranted.

## Conclusion

The current systematic review indicated that there is evidence in favor of using USG for quantitative evaluation of frontal cobb angle in AIS. However, the quality of evidence is low due to high risk of bias and heterogeneity between existing studies. Current literature is insufficient to support the use of USG as a screening and/or follow-up method for AIS. Further investigation addressing the limitations identified in this review is required before USG could be adapted for further clinical use.

## Supplementary Information

Supplementary Material 1.

Supplementary Material 2.

## Data Availability

The datasets used and/or analysed during the current study are available from the corresponding author on reasonable request.

## Abbreviations

PRISMA-DTA: Preferred Reporting Items for Systematic Reviews and Meta-Analyses for Diagnostic Test Accuracy studies
AIS: Adolescent Idiopathic Scoliosis
USG: Ultrasonography
MAD: Mean Absolute Difference
SP: Spinous Process
TP: Transverse Process
SPA: Spinous Process Angle
BMI: Body Mass Index
ICC: Intra-class correlation coefficient
CI: Confidence Interval
QUADAS-2: Quality Assessment of Diagnostic Studies-2
3D: 3 Dimension
