# Supplementary material for: Three-dimensional (3D) ultrasound imaging for quantitative assessment of frontal cobb angles in patients with idiopathic scoliosis – a systematic review and meta-analysis
Source: BMC Musculoskelet Disord. 2025 Mar 5;26:222. doi: 10.1186/s12891-025-08467-5 (PMC11881507; doi:10.1186/s12891-025-08467-5)
Supplement: Supplementary file 1 — Supplementary Material 1. [file 12891_2025_8467_MOESM1_ESM.docx]

**Appendix I. Search Strategy and Terms**

**1. MEDLINE**

Database: Ovid MEDLINE (R) and Epub Ahead of Print, In-Process & Other Non-Indexed Citations

Search Strategy:

1. exp ULTRASONOGRAPHY/ or ultrasonography.mp.
2. (ultrasonogra* or ultra-sonogra*).mp. [mp=title, abstract, original title, name of substance word, subject heading word, floating sub-heading word, keyword heading word, protocol supplementary concept word, rare disease supplementary concept word, unique identifier, synonyms]
3. (ultraso* or ultra-so*).mp. [mp=title, abstract, original title, name of substance word, subject heading word, floating sub-heading word, keyword heading word, protocol supplementary concept word, rare disease supplementary concept word, unique identifier, synonyms]
4. 1 or 2 or 3
5. scoliosis.mp. or exp SCOLIOSIS/
6. spinal curvature.mp. or exp Spinal Curvatures/
7. (scolio* or spin* curv*).mp. [mp=title, abstract, original title, name of substance word, subject heading word, floating sub-heading word, keyword heading word, protocol supplementary concept word, rare disease supplementary concept word, unique identifier, synonyms]
8. 5 or 6 or 7
9. 4 and 8
10. exp Rheumatic Diseases/ or exp Arthritis, Rheumatoid/ or Muscular Diseases/ or exp Arthritis/
11. muscle.mp. or exp Muscles/
12. paraspinal muscle.mp. or exp Paraspinal Muscles/
13. abdominal muscle.mp. or exp Abdominal Muscles/
14. neuromuscular disease.mp. or exp Neuromuscular Diseases/
15. 10 or 11 or 12 or 13 or 14
16. 9 not 15
17. syndrome.mp. or exp SYNDROME/
18. 16 not 17
19. exp ANESTHESIA/ or anesthesia.mp. or exp ANESTHESIA, SPINAL/
20. blood loss.mp. or exp Blood Loss, Surgical/
21. growing rod*.mp. or exp Spinal Fusion/
22. 19 or 20 or 21
23. 18 not 22
24. orthosis.mp. or exp Orthotic Devices/
25. 23 not 24
26. (case report* or case series or case stud*).mp. [mp=title, abstract, original title, name of substance word, subject heading word, floating sub-heading word, keyword heading word, protocol supplementary concept word, rare disease supplementary concept word, unique identifier, synonyms]
27. 25 not 26

**2. EMBASE**

Database: Embase 1910 to Present

Search Strategy:

1. ultrasonography.mp. or exp echography/
2. exp ultrasound/ or exp ultrasound scanner/ or ultrasound.mp.
3. (ultraso* or ultra-so* or ultrasonogra* or ultra-sonogra*).mp.
4. 1 or 2 or 3
5. *idiopathic scoliosis/ or scoliosis.mp. or *adolescent idiopathic scoliosis/ or *scoliosis/
6. spin* curv*.mp. or *Spinal Curvatures/
7. scolio*.mp.
8. 5 or 6 or 7
9. 4 and 8
10. exp syndrome/ or syndro*.mp.
11. 9 not 10
12. (case report* or case stud* or case series).mp.
13. 11 not 12
14. exp anesthesia/ or anesthesia.mp. or exp spinal anesthesia/
15. (blood loss or blood).mp. [mp=title, abstract, heading word, drug trade name, original title, device manufacturer, drug manufacturer, device trade name, keyword, floating subheading word, candidate term word]
16. spinal fusion.mp. or exp spine fusion/
17. 14 or 15 or 16
18. 13 not 17
19. neuromuscular diesease.mp. or exp neuromuscular disease/
20. (nerv* or neuro*).mp. [mp=title, abstract, heading word, drug trade name, original title, device manufacturer, drug manufacturer, device trade name, keyword, floating subheading word, candidate term word]
21. 19 or 20
22. 18 not 21
23. exp skeletal muscle/ or exp muscle/ or muscle*.mp. or exp paraspinal muscle/
24. (prenatal or fetal or fetus).mp. [mp=title, abstract, heading word, drug trade name, original title, device manufacturer, drug manufacturer, device trade name, keyword, floating subheading word, candidate term word]
25. exp "orthopedic prosthesis and orthosis"/ or orthosis.mp. or exp orthosis/
26. (growing rods* or growing-rod*).mp.
27. 23 or 24 or 25 or 26
28. 22 not 27
29. (renal or nephr* or annulus fibrosus).mp. [mp=title, abstract, heading word, drug trade name, original title, device manufacturer, drug manufacturer, device trade name, keyword, floating subheading word, candidate term word]
30. not 29 (186)

**3. CINAHL**

Database: CINAHL

Search Strategy:

S1 (MH "Ultrasonography+") OR "ultrasonography"

S2 "ultrasound" OR (MH "Ultrasonography+")

S3 "ultrasound imaging"

S4 "ultrasound or sonography or sonogram or ultrasonography"

S5 S1 OR S2 OR S3 OR S4

S6 (MH "Scoliosis+") OR "scoliosis" OR (MH "Scoliosis, Idiopathic, Adolescent") OR (MH "Spinal Curvatures+")

S7 S5 AND S6

S8 (MH "Anesthesia+") OR "anesthesia" OR (MH "Anesthesia, Spinal")

S9 S7 not S8

S10 (MH "Orthoses Fitting") OR (MH "Orthoses Design") OR (MH "Orthoses+") OR "orthosis"

S11 S9 not S10

S12 "nerve"

S13 S11 not S12

**4. CENTRAL**

Database: CENTRAL (Cochrane Central Register of Controlled Trials) Library

Search Strategy:

#1 ultrasonography

#2 ultrasound

#3 ultrasonogra* or ultra-sonogra* or ultraso* or ultra-so*

#4 #1 or #2 or #3

#5 scoliosis

#6 spinal curvature

#7 scolio* or spin* curv*

#8 #5 or #6 or #7

#9 #4 and #8

#10 anesthesia

#11 #9 not #10
