# Supplementary material for: Three-dimensional (3D) ultrasound imaging for quantitative assessment of frontal cobb angles in patients with idiopathic scoliosis – a systematic review and meta-analysis
Source: BMC Musculoskelet Disord. 2025 Mar 5;26:222. doi: 10.1186/s12891-025-08467-5 (PMC11881507; doi:10.1186/s12891-025-08467-5)

**Appendix II. Template of Quality Assessment of Diagnostic Studies (QUADAS-2) quality appraisal tool**


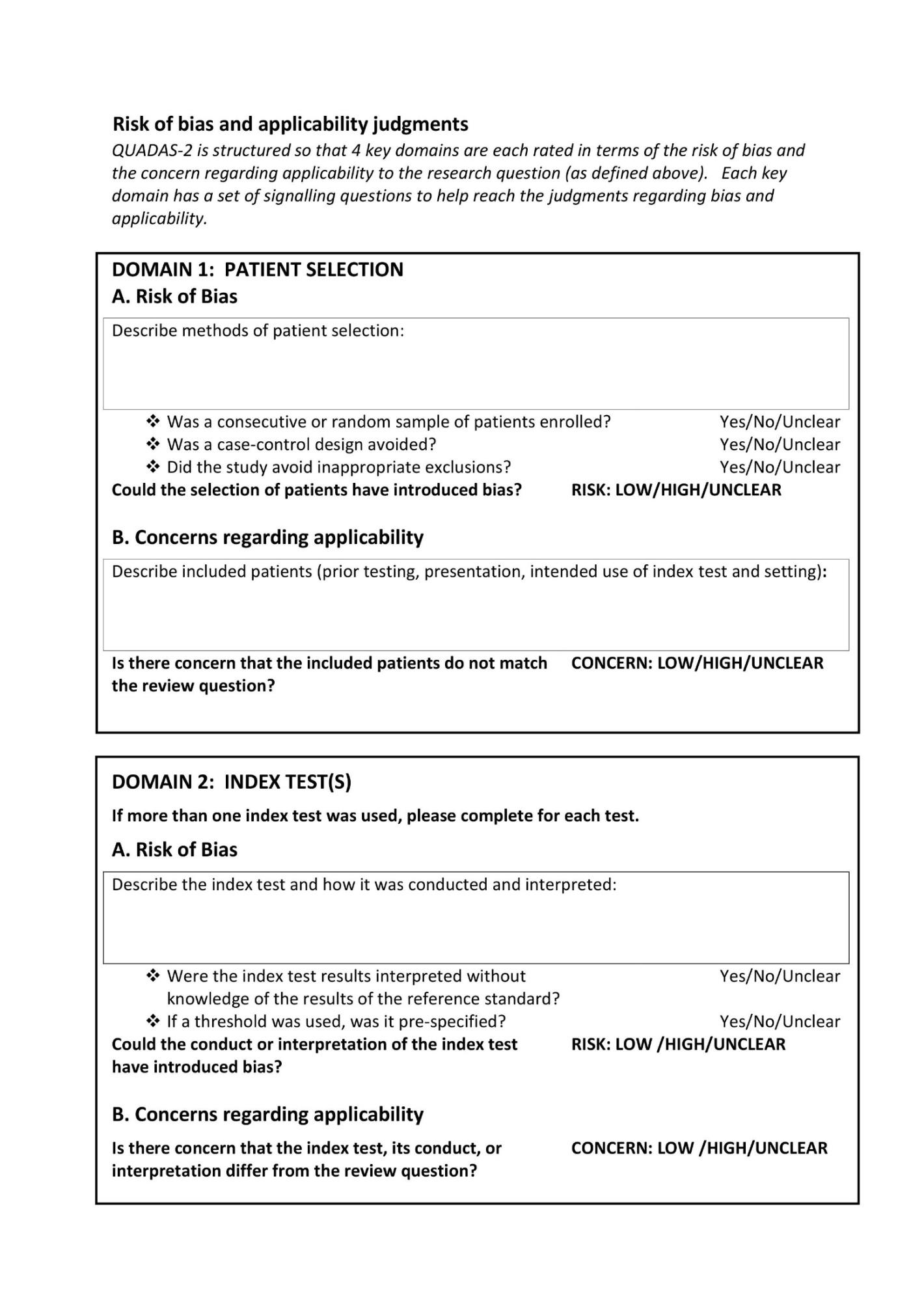


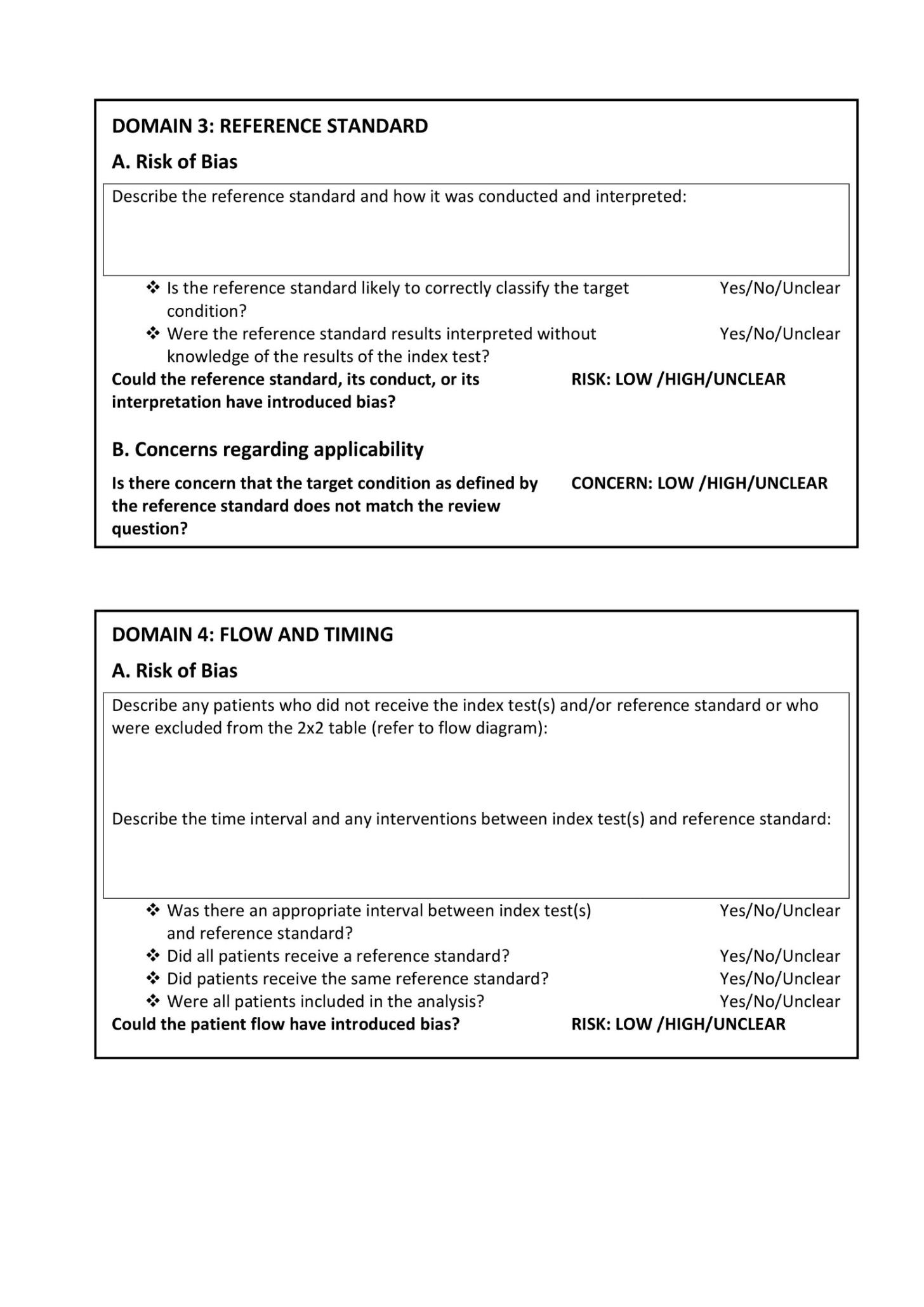

Supplement: Supplementary file 2 — Supplementary Material 2. [file 12891_2025_8467_MOESM2_ESM.docx]
